# Materia Medica in Rhyme

**Published:** 1846-03

**Authors:** 


*Materia Medica in Rhyme.*—A warm advocate for the Thomsonian practice, has communicated a poem to the Botanico-Medical Recorder, that must have been taken with a wry mouth by the editor. However, he evidently wished to oblige a poet who sings on the major key in praise of a system that is invariably lauded in proportion to one's ignorance. Here is a specimen.

"Botanic remedies were designed,
To heal the body and soothe the mind.
Let every tongue and every pen,
Proclaim the virtues of cayenne.
Nor will we fear to use it freely;
Nor value less the good lobelia."

*Ibid.*

